# Vaccine innovation prioritisation strategy: Findings from three country-stakeholder consultations on vaccine product innovations

**DOI:** 10.1016/j.vaccine.2021.08.024

**Published:** 2021-12-03

**Authors:** Mercy Mvundura, Collrane Frivold, Anna Janik Osborne, Priyanka Soni, Joanie Robertson, Sandeep Kumar, Jacqueline Anena, Abdoulaye Gueye, Marion Menozzi-Arnaud, Birgitte Giersing, Anna-Lea Kahn, Tiziana Scarna, Debra Kristensen

**Affiliations:** a PATH, 2201 Westlake Avenue, Suite 200, Seattle, WA 98121, USA; b Gavi, the Vaccine Alliance, Global Health Campus, Chemin du Pommier 40, 1218 Grand-Saconnex, Geneva, Switzerland; c PATH, 15th Floor, Dr. Gopal Das Bhawan, 28 Barakhamba Road, Connaught Place, New Delhi 110001, India; d PATH, PO Box 7404, Kampala, Uganda; e PATH, Fann Residence, Rue Saint-John Perse Angle F, Dakar, Senegal; f Vaccine Product & Delivery Research, Immunisation, Vaccines and Biologicals, World Health Organization, CH-1211 Geneva 27, Switzerland

**Keywords:** Vaccine product innovation, Immunization, Vaccine, Low- and middle-income countries, Prioritization, Delivery technologies, Product development, Coverage and equity, CHAI, Clinton Health Access Initiative, CPADs, compact, prefilled, autodisable devices, CTC, controlled temperature chain, EPI, Expanded Programme on Immunization, HPV, human papillomavirus, IPV, inactivated poliovirus vaccine, MAPs, microarray patches, SIP, sharps injury protection, SDI, solid dose implants, VIPS, Vaccine Innovation Prioritisation Strategy, WHO, World Health Organization, UNICEF, United Nations Children’s Fund, VVM-TIs, vaccine vial monitors with threshold indicators

## Abstract

As part of the Vaccine Innovation Prioritisation Strategy (VIPS), three immunization-stakeholder consultations were conducted between September 2018 and February 2020 to ensure that countries’ needs drove the prioritization of vaccine product innovations.

All consultations targeted respondents with immunization program experience. They included: (1) an online survey to identify immunization implementation barriers and desired vaccine attributes in three use settings, (2) an online survey to identify and evaluate the most important immunization challenges for ten exemplar vaccines, and (3) in-depth interviews to better understand the perceived programmatic benefits and challenges that could be addressed by nine innovations and to rank the innovations that could best address current challenges.

The first consultation included responses from 442 participants in 61 countries, representing 89% of the 496 respondents who correctly completed at least one section of the online survey. For facility-based settings, missed opportunities for vaccination due to reluctance to open multidose vaccine vials was the barrier most frequently selected by respondents. In community-based (outreach) and campaign settings, limited access to immunization services due to geographic barriers was most frequently selected. Multidose presentations with preservative or single-dose presentations were most frequently selected as desired vaccine attributes for facility-based settings while improved thermostability was most frequently selected for outreach and campaign settings. The second online survey was completed by 220 respondents in 54 countries. For the exemplar vaccines, vaccine ineffectiveness or wastage due to heat or freeze exposure and missed opportunities due to multidose vial presentations were identified as the greatest vaccine-specific challenges. In-depth interviews with 84 respondents in six countries ranked microarray patches, dual-chamber delivery devices, and heat-stable/controlled temperature chain qualified liquid vaccines as the three innovations that could have the greatest impact in helping address current immunization program challenges.

These findings informed the VIPS prioritization and provided broader application to designing immunization interventions to better meet country needs.

## 1 Introduction

Immunization programs in low- and middle-income countries face challenges with current vaccine products, such as the need for refrigerated storage and transport, complex preparation and administration requirements, and multidose container presentations; these challenges can lead to higher vaccine wastage, safety issues, and missed vaccination opportunities [1], [2]. Global immunization coverage has plateaued over the last decade. Despite the fact that as a result of population growth, more children than ever are receiving three doses of diphtheria, tetanus, and pertussis vaccine before their first birthday, in 2019 there were at least 20 million children who were un- or under-vaccinated [3], [4]. There is increasing recognition of the need to employ targeted solutions to extend vaccine access to reach the unreached and increase equitable coverage of vaccines [5]. The global COVID-19 crisis has further highlighted the need for vaccine product innovations that enable vaccines to reach underserved populations, particularly during rapid, large-scale responses. Vaccine product innovations (e.g., on primary containers, delivery technologies, heat-stable and freeze-stable formulations, packaging, labeling, and supply systems technologies) are powerful tools that could help overcome vaccine coverage and equity shortfalls. Such innovations have the potential to simplify logistics, increase the acceptability and safety of immunization, minimize missed opportunities, and facilitate outreach of vaccines [2], [5], [6].

In the *Gavi, Vaccine Alliance (Gavi) 2016*–*2020 Supply and Procurement Strategy*, the need to drive product innovation to better meet country needs and support Alliance goals on coverage and equity was defined as one of the strategic priorities to create healthy markets for vaccines and other immunization products in the countries Gavi supports [7]. Under this priority, a key activity was alignment of partners and setting a common agenda on vaccine product innovation. To lead this effort, the Vaccine Innovation Prioritisation Strategy (VIPS) was launched in 2017 by Gavi, the World Health Organization (WHO), Bill & Melinda Gates Foundation, United Nations Children’s Fund (UNICEF), and PATH—known collectively as the VIPS Alliance [8].

At its inception, the goal of VIPS was to articulate a clear and aligned perspective on vaccine product priority innovations and communicate these priorities to donors, immunization program partners, as well as technology and vaccine developers, to help inform priority setting and investment decisions. This goal was achieved in May 2020 on the completion of a comprehensive evaluation process, which culminated in the prioritization of three innovative vaccine technologies: microarray patches (MAPs), heat-stable and controlled temperature chain (CTC) qualified vaccines, and barcodes on primary packaging. The prioritized technologies represent a diversified portfolio with innovations at varying stages of the product development pathway and addressing different programmatic challenges. Details on the innovations evaluated as well as the methodology and process leading to the prioritization is described elsewhere [8] and summarized in the accompanying article, *A Global Collaboration to Advance Vaccine Product Innovations – the Vaccine Innovation Prioritisation Strategy* [9]. Briefly, the VIPS prioritization process consisted of two phases, of which the first began in April 2018 and evaluated 24 innovation types. These 24 innovations were assessed for their ability to address general immunization program challenges, their applicability to one or more vaccines, and their potential impacts on health, coverage and equity, safety, and economic costs in comparison to current technologies in use. This first evaluation phase resulted in a shortened list of nine innovation types that were assessed to have attributes that offered the greatest potential public health value. These nine innovations were further analyzed against a specific set of representative vaccine antigens during a second evaluation phase, occurring between June 2019 and May 2020. In this second phase, each innovation was assessed in combination with the vaccines it could apply to and evaluated against the vaccine-specific challenges it could address; its potential impact on health, coverage and equity, safety, economic costs, and environment; as well as technical readiness and commercial feasibility. Innovations that apply to all vaccines were also evaluated using similar criteria. The VIPS process involved in-depth consultations with a diverse set of country- and global-level stakeholders, including industry and regulators. It also involved the development and application of a qualitative analytical framework capable of evaluating a variety of technologies at different stages along the product development continuum from technology ideation to implementation.

Establishing a better understanding of countries’ needs was intended as the foundation of VIPS. As such, between 2018 and 2020 the VIPS Alliance conducted three consultations with varied country decision-makers and Expanded Programme on Immunization (EPI) staff to inform the prioritization process. Opinions from these stakeholders collected through the consultations were critical inputs used for that process. This article describes the methodology used, the results, and conclusions from these three country-stakeholder consultations.

## 2 Materials and methods

The surveys and interview tools underwent pre-testing by potential respondents prior to being finalized and used. No incentives were provided to the respondents for participation in the consultations. Every effort was made to obtain maximum geographic and economic diversity in the responses, ensuring countries from a broad range of regions and income levels were targeted for participation. The results from all consultations were analyzed in Microsoft Excel.

### 2.1 Online survey on general immunization barriers

The first stakeholder consultation was conducted between September 2018 and January 2019 to identify general immunization implementation barriers (i.e., across vaccine types, formulations, and presentations, and not specific to a certain vaccine) that could be addressed by vaccine product innovations. The target audience for the survey was EPI managers, procurement staff, logistics/supply chain staff, data managers, senior policymakers (including National Immunization Technical Advisory Groups), health care service providers, implementing partners (nongovernmental organizations, civil society organizations), UNICEF and WHO country/regional office staff, and in-country research/university partners. This consultation was carried out by means of an online survey offered in four languages (i.e., English, French, Spanish, Russian), which was widely distributed via online professional forums, relevant networks across all WHO regions, and targeted emails to potential respondents including vaccine-focused distribution lists (i.e., TechNet-21, BID Learning Network, and Africa Resource Centre) [10], [11], [12]. Clinton Health Access Initiative (CHAI) staff facilitated completion of the survey by health care service providers without internet access in Uganda and Kenya.

The survey asked each respondent to select, from a list of 18 implementation barriers, the 5 they thought were most important in preventing improvements in vaccine coverage and equity. Respondents were asked to select the barriers in the context of three vaccine use settings: routine facility-based immunization, routine community-based (outreach) immunization, and campaigns including outbreak response. A second question asked them to select 5 out of a list of 15 vaccine product attributes, which they thought could best help address the identified implementation barriers in the same use settings. The pre-populated lists of country implementation barriers and vaccine product attributes given to respondents in this survey were developed through literature review and expert inputs by VIPS Alliance members; only barriers that could be addressed by vaccine product innovations, and similarly only vaccine products attributes that could address the barriers, were included in the list (e.g., barriers related to immunization financing were not included). Information was also collected through open-ended questions on additional barriers and desirable vaccine product attributes. See Supplementary Table 1 for detailed survey questions.

The survey responses on the implementation barriers and vaccine product attributes were analyzed by use setting. Due to a software issue with the online survey, some respondents selected more than five barriers or vaccine attributes per setting. Therefore, we excluded the data of those respondents who provided more than five barriers or vaccine product attributes for the use setting being evaluated in this analysis. The ranking of implementation barriers and the vaccine product attributes was then compared to evaluate whether the key implementation barriers selected by respondents could be addressed by the most frequently selected vaccine product attributes.

### 2.2 Online survey on vaccine-specific immunization challenges

A second online survey was conducted between November 2019 and February 2020 to identify vaccine-specific immunization challenges that could be addressed by the nine innovations short-listed by VIPS. The survey was conducted in five languages (i.e., English, French, Spanish, Portuguese, Russian). Immunization experts with knowledge of vaccination strategies and existing vaccine products from Gavi-supported and non-Gavi-supported countries were invited by email to complete this online survey. The survey was shared with potential respondents through distribution lists of country immunization experts managed by Gavi, PATH, CHAI, and WHO regional and country offices.

The questions in this second online survey focused on ten exemplar vaccines, which were identified as part of the second evaluation phase of VIPS [8]. These vaccines were selected to be representative of the broader vaccine landscape based on vaccine type, formulation, and presentation. During the survey design, an initial list of challenges was provided for each of the ten vaccines, based on the priority immunization implementation barriers identified in the first survey. These initial lists were then further refined through consultation with vaccine delivery program experts at WHO. When completing the survey, respondents were asked for inputs concerning only the vaccines that they had experience with. For each vaccine evaluated, the respondent had to select challenges from the list provided that applied to the vaccine; they also had the opportunity to suggest additional challenges not included in the provided list. Then, from the challenges they had selected (including the ones added), the respondent was asked to short-list and rank the three most important ones. If the respondent identified fewer than three challenges for the vaccine, they were asked to rank all the challenges they had identified. Supplementary Table 2 shows the second online survey questionnaire.

Given that barcodes address a unique set of challenges compared to other innovations evaluated which are focused on vaccine preparation and administration challenges, the survey included separate questions that informed the evaluation of barcodes. These questions focused on electronic systems for vaccine inventory and electronic patient record keeping in order to gather data on current use of electronic systems as well as country interest and readiness to use barcodes on primary containers. These questions are also shown in Supplementary Table 2.

During data analysis, we tabulated by vaccine the number of respondents who selected a given challenge as one of their three most important challenges. We included responses from respondents who selected at least one and up to three of the challenges for any of the vaccines. We also tabulated the responses from the questions on electronic systems.

### 2.3 In-depth interviews to evaluate VIPS short-listed innovations

The third country consultation took place between November 2019 and February 2020, in parallel to the second online survey and consisted of in-person interviews. These interviews were conducted in six countries in Africa and Asia to gather feedback from decision-makers and immunization staff on the nine short-listed VIPS innovations. The countries included in the consultations were based on the availability of PATH and CHAI staff to conduct the interviews and willingness and availability of country stakeholders to participate. The nine short-listed innovations of focus in this consultation were classified as either vaccine-specific (i.e., applicability is vaccine dependent) or vaccine-agnostic (i.e., relevant to all vaccines). The vaccine-specific innovations were compact, prefilled, autodisable devices (CPADs), dual-chamber delivery devices, MAPs, solid dose implants (SDIs), freeze damage resistant liquid vaccines, and heat-stable/CTC qualified liquid vaccines. Vaccine-agnostic innovations were sharps injury protection (SIP) syringes, combined vaccine vial monitors with threshold indicators (VVM-TIs), and barcodes on vaccine primary containers. For each innovation, the aim was to understand the perceived benefits of the innovation and challenges that could hinder the adoption of the innovation, specific vaccines for which the innovation would be most useful, as well as interest in eventual adoption and use. Each respondent was also asked to select the three innovations they thought would have the greatest impact in helping address their immunization program’s current needs and priorities.

Interview respondents were purposively selected because they were known to have experience with, and knowledge of, immunization systems and strategies as well as vaccine management. These respondents were selected according to two profiles: the first group consisted of those with decision-making authority or influence over vaccine purchase decisions (referred to as decision-makers). This group included EPI program managers at the national and regional levels, and advisors for the EPI, such as members of National Immunization Technical Advisory Groups and Interagency Coordinating Committees. The second group consisted of immunization staff working within the national programs whose roles included managing and administering vaccines at the district or health facility levels.

The in-depth interviews were conducted by PATH and CHAI staff who coordinated with the EPI managers in each country to identify the respondents for the survey, using the participant inclusion criteria outlined above. Interviewers were trained beforehand to ensure consistency in conducting the interviews. Ministries of health in each country approved the in-depth interviews. The PATH Research Determination Committee determined that this activity did not meet the definition of research involving human subjects so the survey did not require an ethical approval.

Before answering questions on each innovation, the respondent was familiarized on the use of the innovation without being provided with information on potential benefits and challenges. Where applicable, commercially available examples or prototypes of the innovation were shown to the respondent. Technology cards were also presented with images that described the purpose of each innovation and how it is used. A short video clip was then shown to demonstrate the use of some of the technologies where their use was not deemed intuitive based on the description provided. After the familiarization with each innovation, the interviewer asked semi-structured, open-ended questions with a research approach to questioning, to engage the respondent in conversation, exploring the anticipated benefits and trade-offs of the innovation based on the respondent’s experience and opinions. The respondent provided their views on the benefits and challenges of the innovation and the interviewer probed to inquire if there were more benefits or tradeoffs the respondent wanted to provide but not to lead them towards specific benefits or tradeoffs. If after this probing, the respondent said they had no additional benefits or tradeoffs to mention, the interviewer moved to the next question, irrespective of the number of benefits or tradeoffs that had been mentioned by the respondent. Additionally, for innovations that are vaccine-specific, the respondent was asked to provide examples of vaccines they believed could benefit from their use. This process was repeated until the respondent had evaluated each of the nine innovations. After the evaluation of the last innovation, the respondent was asked to select and rank the three most preferred innovations based on what they believed would have the greatest impact in helping address their immunization program’s current challenges. The questions used in the interviews are shown in Supplementary Table 3.

Four different orders of presenting the innovations were used and rotated between interview participants to avoid biasing the quality of responses through interview fatigue. Responses were documented on tablets or smartphones using Open Data Kit software [13]. The interviews were audio-recorded with permission of respondents to allow checking of the accuracy of data entry after the interviews were completed. For ease of data entry during the interviews, anticipated benefits and challenges were available to interviewers to select in the Open Data Kit interface, along with space to enter additional comments provided by the respondents. The respondents could not see this interface. Vaccines that could benefit from use with each innovation were also pre-populated in the data form with additional spaces provided to allow entry of other vaccines that might be mentioned by the respondents.

During data analysis for each innovation, the number of respondents stating each benefit and challenge were counted as well as the vaccines for which the innovation would be particularly useful. Data were aggregated and analyzed for all countries. The results were also disaggregated based on roles (immunization staff vs. decision-makers). The overall ranking of innovations, based on the innovations that respondents believed would have the greatest impact in helping address their immunization program’s current challenges, was achieved using a weighted scores approach. For the weighted scores, if the innovation was ranked as a first choice, it was given a weight of 3 points, a second choice was given a weight of 2 points, and a third choice was given a weight of 1 point. A weighted scores approach was used for ranking innovations given that all respondents had selected their top three innovations and ranked them by order of anticipated impact.

## 3 Results and discussion

### 3.1 Online survey on general immunization barriers

The first online survey was completed by 496 individuals, of which 442 (89%) correctly selected at most five barriers or vaccine product attributes, per the survey instructions, for at least one of the delivery settings. These 442 respondents were from 61 Gavi-supported and non-Gavi-supported countries. Seventy five percent of these respondents were from Gavi-supported African countries. Eighty percent of the countries represented in the survey had less than 10 respondents. The summary of survey respondents by organization is presented in Supplementary Fig. 1 and shows that the majority of respondents (55 percent) were ministry of health staff at different levels of the health system including the service delivery level.

For each setting (i.e., routine facility-based, outreach, and campaigns) a total of 268, 254, and 298 respondents, respectively, selected at least one to at most five of the most important barriers preventing improvements in immunization coverage. The number of respondents selecting each of the barriers for each use setting are shown in Table 1. Missed opportunities for vaccination due to reluctance to open multidose vials was the barrier selected by the most respondents (126/268) for routine facility-based immunization. Limited access to immunization services due to geographic barriers (e.g., remote populations) was selected by the most respondents as the greatest barrier for both outreach and campaign settings, 147/254 and 126/298, respectively. There were broader parallels in the priority implementation barriers between outreach and campaign settings, because for both settings, social barriers (e.g., limited access to immunization services for marginalized populations, such as those living in urban slums, single mothers, orphans and vulnerable children, certain ethnic/religious groups, refugees, etc.) was the second-most selected. For routine facility-based settings, inadequate infrastructure (e.g., buildings and electricity) for vaccine and immunization equipment storage at delivery points was the second-most selected barrier.

**Table 1:** Number of respondents selecting each implementation barrier to improved vaccine coverage and equity by use setting—first online survey.

	Facility-based (n = 268)	Outreach (n = 254)	Campaigns (n = 298)
Vaccine product damage during transport and delivery (e.g., glass vial breakage)	28	62	67
Supply shortages and wastage due to poor tracking of shipments and poor monitoring of inventory	96	55	57
Inadequate infrastructure (e.g., buildings and electricity) for vaccine and immunization equipment storage at delivery points (excluding cold chain space)	108^b^	61	68
Supply shortages due to insufficient cold chain space for vaccine storage	65	42	70
Supply shortages for commodities needed to administer a vaccine (e.g., diluents, injection and reconstitution syringes, sharps disposal containers, disinfectants)	56	38	36
Wastage due to exposure (or possible exposure) to heat	22	62	92
Wastage due to exposure (or possible exposure) to freezing	26	13	18
Damage due to inappropriate storage conditions (e.g., unreadable labels from humidity or mold exposure)	36	41	62
Limited access to immunization services due to geographic barriers (e.g., remote population)	100	147^a^	126^a^
Limited access to immunization services due to social barriers (e.g., marginalized populations that require greater outreach such as urban slums, single mothers, orphans and vulnerable children, certain ethnic/religious groups, refugees, IDPs, migrants, people living with HIV, etc.)	82	121^b^	125^b^
Limited access to immunization services due to financial barriers faced by patients/caregivers (e.g., out-of-pocket expenditure for transport; loss of daily wages; cost of vaccination card)	71	49	27
Mistrust in skills and/or intentions of health care service providers (e.g., lack of confidence in the reliability and competence of health workers skills)	24	44	93^c^
Fear of injections and needles (especially in case of multiple injections)	64	60	59
Discomfort after vaccination (e.g., pain or swelling at site of administration)	69	54	81
Lack of available health care service providers leading to missed opportunities due to overburdened services	101^c^	110^c^	65
Lack of appropriate training and skills leading to vaccine misuse or missed opportunities due to errors during service delivery (e.g., errors in reconstitution, administration)	75	55	85
Missed opportunities or vaccine misuse due to complexity of vaccine preparation or administration procedures (e.g., easier to administer vaccines could increase coverage for birth dose testing)	35	27	26
Missed opportunities due to reluctance to open multidose vials of vaccines without preservative	126^a^	81	37

The values in this table are the number of respondents selecting each implementation barrier as one of the top five implementation barriers out of the barriers provided by use setting: routine facility-based immunization, outreach, campaigns. The survey was correctly completed by 442 respondents but not all respondents provided responses for each survey section focused on immunization barriers or vaccine attributes) and for each use setting. As a result, the number of respondents included in each sub-analysis is different.

We indicate the barriers that were selected by most respondents using this key:

a Barrier selected by most respondents for the use setting.

b Barrier selected by second-most respondents for the use setting.

c Barriers selected by third-most respondents for the use setting.

Similarly, for each setting (i.e., routine facility-based, outreach, and campaigns) a total of 309, 306, and 324 respondents, respectively, selected at least one to at most five vaccine product attributes that could help address the implementation barriers. The number of respondents selecting each vaccine product attribute are shown in Table 2. Prevention of missed opportunities (e.g., through multidose presentation with preservative or single-dose presentation) was selected by the most respondents (223/309) as the desirable product attribute for routine facility-based settings, which aligns with missed opportunities being an implementation barrier selected by the most respondents for this setting (as per Table 1). The ability to withstand heat exposure was selected by the most respondents as the desired attribute to meet challenges faced for outreach and campaigns (176/306 and 197/324, respectively). Such an attribute could enable vaccines to reach populations that typically have limited access to immunization services due to geographic barriers. Therefore, the desired vaccine attributes identified by survey respondents align with the barriers they most frequently selected.

**Table 2:** Number of respondents selecting each desired vaccine attribute by use setting—first online survey.

	Facility-based (n = 309)	Outreach (n = 306)	Campaigns (n = 324)
Ability to withstand heat exposure	125	176^a^	197^a^
Ability to withstand freeze exposure	99	49	44
Delivery aligned with existing immunization schedules or with other health commodities	152^b^	113	58
Suitable for use in controlled temperature chain (CTC)	108	86	84
Suitable for administration by lesser trained personnel	79	121^c^	163^b^
Suitable for self-administration (or administration by caregiver)	30	58	53
Vaccine product that helps prevent missed opportunities (e.g., multidose presentation with preservative or single-dose presentation)	223^a^	174^b^	103
Minimal number of separate components necessary for vaccine delivery	74	84	86
Acceptable to patients/caregivers (e.g., reduced fear of pain through delivery without needles)	132^c^	117	122
Reduced risk of incorrect preparation	53	49	63
Reduced risk of vaccine contamination	40	61	76
Reduced risk of incorrect delivery	45	44	62
Reduced risk of needle-stick injury	38	39	53
Reduced space required for storage and transport	103	92	94
Easier to use, leading to reduced time by vaccinators to prepare and administer the vaccine	126	114	146^c^

The values in this table are the number of respondents selecting each desired vaccine attribute as one of the top five desired vaccine attributes out of the attributes provided by use setting: routine facility-based immunization, outreach, campaigns. The survey was correctly completed by 442 respondents but not all respondents provided responses for each survey section focused on immunization barriers or vaccine attributes) and for each use setting. As a result, the number of respondents included in each sub-analysis is different.

We indicate the vaccine attributes that were selected by most respondents using this key:

a Vaccine attribute selected by most respondents for the use setting.

b Vaccine attribute selected by second-most respondents for the use setting.

c Vaccine attribute selected by third-most respondents for the use setting.

In addition to the attributes listed in the survey, several vaccine product attributes were mentioned as desirable by survey respondents. These included needle-free vaccine presentations (i.e., oral, nasal spray, MAPs, aerosols), combination/multiple antigen vaccines, reducing the number of doses in the regimen/vaccine schedule, and improved thermostability including shelf-stable vaccine products that do not require cold chain storage.

### 3.2 Online survey on vaccine-specific immunization challenges

The second survey was completed by 220 stakeholders from 54 countries including global- and regional-level stakeholders. The global- and regional-level stakeholders accounted for 26 percent of the respondents and about half of the respondents were from Gavi-supported African countries. Of the countries with respondents participating in the survey, 85 percent had less than 10 respondents completing the survey. See Supplementary Figure 2 for a summary of survey respondents by organization.

Stakeholder rankings of vaccine-specific challenges are shown in Table 3. As seen in Table 3, fewer responses were received for newer vaccines and vaccines used in specific regions (such as vaccines against yellow fever, rabies and typhoid) as the survey guided participants to only provide responses for those vaccines with which they had experience. Vaccine ineffectiveness or wastage due to damage by freeze exposure was the most frequently selected challenge for pentavalent, inactivated polio, human papillomavirus (HPV), and hepatitis B birth dose vaccines. Vaccine ineffectiveness or wastage due to heat exposure was the most frequently selected challenge for measles-containing, rotavirus, typhoid conjugate, and rabies vaccines. Both challenges (vaccine ineffectiveness or wastage due to either heat or freeze exposure) were selected as the top two challenges for five of the ten vaccines evaluated.

**Table 3:** Number of respondents selecting each challenge as one of the three most important challenges facing delivery of the priority representative vaccines—second online survey.

Challenge for the vaccine	Penta (n = 155)	MCV (n = 119)	IPV (n = 103)	Rota (liquid) (n = 64)	HPV (n = 49)	HepB BD (n = 51)	YF (n = 57)	MenA (n = 54)	TCV (n = 14)	Rabies (n = 16)
Vaccine ineffectiveness/wastage due to freeze exposure	102^a^		56^a^	25^b^	19^a^	40^a^		8	5^b^	
Vaccine ineffectiveness/wastage due to heat exposure	70^b^	69^a^	51^b^	31^a^	12	21^b^		18	7^a^	7^a^
Vaccine wastage or missed opportunities due to multidose vial		66^b^	12	3	4		32^a^	22^a^	5^b^	3
Reduced acceptability due to painful administration	62^c^	12	21	1	15^b^	7	11	7	3	6^c^
Reconstitution-related safety issues		54^c^					30^b^	17^c^		3
Cold chain requirements during outreach	40	32	24^c^	19^c^	15^b^	16^c^	17^c^	20^b^	3	1
Contamination risk due to multidose vial	34		15	0	1	7			0	
Negative impact of the environment due to waste disposal practices	32	17	21	17	6	5	11	7	0	0
Needle-stick injuries	25	14	8		7	3	16	13	1	4
Difficult to deliver vaccine to correct injection depth	6	5	2		1	1	1	2	2	3
Difficult preparation requiring trained personnel	2	15	2	1	1	6	9	9	4	7^a^

Abbreviations: HepB BD, hepatitis B birth dose; HPV, human papillomavirus; IPV, inactivated poliovirus vaccine; MCV, measles-containing vaccine; MenA, meningococcal group A; penta, pentavalent (DTP-HepB-Hib); rota, rotavirus; TCV, typhoid conjugate vaccine; YF yellow fever.

The values in this table are the number of respondents selecting each vaccine attribute as one of the three important challenges facing delivery of the priority representative vaccines. The survey was correctly completed by 220 respondents but not all respondents provided responses for each vaccine as they were instructed to only provide responses for the vaccines with which they have experience. As a result, the number of respondents (n) included in each vaccine analysis is different. Also, some respondents only included one or two challenges, while others provided up to three as requested.

We indicate the challenges to the vaccine that were selected by most respondents using this key:

a Challenge for the vaccine selected by most respondents.

b Challenge for the vaccine selected by second-most respondents.

c Challenge for the vaccine selected by third-most respondents.

A subset of respondents answered the questions included to provide information to evaluate the barcode innovation and they reported that in the public immunization system in the countries where they primarily work, 58 percent (75/130) currently use an electronic system for vaccine inventory and 22 percent (28/128) for patient records. Of the respondents who indicated that the public immunization program in their country does not currently use electronic systems or they do not know, 91 percent (50/55) responded that transitioning to an electronic system would be beneficial to vaccine inventory, and 92 percent (92/100) responded that it would be beneficial to patient vaccination records. These results suggest that there is strong interest from survey respondents in electronic systems, for which barcodes could improve accuracy of data entry. However, to realize the full potential offered by barcodes on primary packaging, a transition to use of electronic inventory and health records would be required down to the health facility level, which could be a challenging process in many low- and middle-income countries because of the equipment costs, training needs, and other requirements.

This second survey identified the immunization challenges that apply to exemplar vaccines, providing insight on which vaccine product attributes might offer broad cross-vaccine benefits. Some vaccine-specific challenges selected across multiple assessed vaccines are consistent with the generic barriers most selected in the first survey (e.g., vaccine ineffectiveness/wastage due to heat exposure) while others (e.g., vaccine ineffectiveness/wastage due to freeze exposure) were not strongly highlighted by the first survey. The results also informed the assessment of barcodes and showed while some countries have initiated the transition to electronic inventory management, even fewer of them have initiated the transition for electronic patient records.

### 3.3 In-depth interviews to evaluate VIPS short-listed innovations

A total of 84 respondents were interviewed across six countries: Ethiopia (n = 15), Nepal (n = 15), Nigeria (n = 21), Senegal (n = 15), Uganda (n = 17), and Mozambique (n = 1). A total of 55 immunization staff and 29 decision-makers completed the surveys.

#### 3.3.1 Perceived benefits of the innovations

Table 4, Table 5 show the perceived benefits mentioned for each of the nine short-listed innovations. For the vaccine-specific innovations, easier preparation or easing of logistics was the most frequently mentioned benefit identified for CPADs (75/84 respondents), dual-chamber delivery devices (71/84), MAPs (76/84), and SDIs (66/84), as shown in Table 4. Most respondents (78/84) identified the benefit of freeze damage resistant liquid vaccines as preventing vaccine damage/vaccine wastage due to accidental freezing. For CTC qualified liquid vaccines, 56/84 respondents mentioned allowing vaccines to be kept out of the cold chain as a benefit while 55/84 respondents mentioned preventing vaccine damage/vaccine wastage due to suspected heat exposure as a benefit. There was general consistency in the types of perceived benefits mentioned by decision-makers and immunization staff but there were some differences in the rankings between these two groups. For example, for MAPs, improving ease of use was the most mentioned benefit by immunization staff while increased acceptability to vaccine recipients or caregivers was the most mentioned benefit by decision-makers.

**Table 4:** Perceived benefits identified for the vaccine-specific innovations and number and percentage of respondents mentioning the benefits of each innovation—in-depth interviews of 84 total respondents composed of 55 immunization staff (IS) and 29 decision-makers (DM).

Potential benefit	Compact, prefilled, autodisable devices			Dual-chamber delivery devices			Microarray patches			Solid dose implants			Freeze damage resistant liquid vaccines			Heat-stable/CTC qualified liquid vaccines		
	n = 84	% of IS	% of DM	n = 84	% of IS	% of DM	n = 84	% of IS	% of DM	n = 84	% of IS	% of DM	n = 84	% of IS	% of DM	n = 84	% of IS	% of DM
Easier to prepare and or use/eases logistics	75^a^	100%	59%	71^a^	96%	62%	73^a^	100%	62%	66^a^	95%	48%	15^c^	22%	10%			
More acceptable to vaccine recipients or caregivers	24	31%	24%	21	27%	21%	64^b^	82%	66%	54^b^	64%	66%						
Saves health care workers time	42^c^	60%	31%	46	65%	34%	43^c^	58%	38%	42^c^	58%	34%	28^b^	33%	34%	22	27%	24%
Reduces needle-stick injuries							38	49%	38%	32	40%	34%						
Reduces vaccine contamination/use of wrong diluent (for reconstituted vaccines only)	41	55%	38%	47^c^	65%	38%	28	38%	24%	22	35%	10%						
Improves vaccine coverage or vaccine reach										14	11%	28%	2	4%	0%	22	27%	24%
Decreases vaccine wastage	50^b^	64%	52%	54^b^	65%	62%	24	31%	24%	26	35%	24%						
Enables delivery outside of a health facility/by less skilled personnel	14	15%	21%				21	18%	38%							46^c^	64%	38%
Helps prevent missed opportunities	19	22%	24%	22	25%	28%	19	25%	17%									
Reduces adverse events following immunization							18	22%	21%				3	2%	7%	3	5%	0
Improves delivery of the correct dose amount	37	55%	24%	33	45%	28%				13	16%	14%						
Improves vaccine coverage	17	18%	24%	17	16%	28%	24	31%	24%									
Prevents vaccine damage/vaccine wastage due to suspected freezing													78^a^	96%	86%	16	22%	14%
Prevents vaccine damage/vaccine wastage due to suspected heat exposure				14	24%	3%										55^b^	71%	55%
Allows vaccine to be kept out of the cold chain/reduces cold chain logistics																56^a^	80%	41%
Reduces the need for buying vaccine refrigerators/saves electricity																3	0%	10%
Improves delivery to the correct injection depth				13	16%	14%												
Improves waste disposal/reduce health care waste	21	24%	28%							17	22%	17%						
Improves potency/quality													15	20%	14%	3	5%	0%
Improves timeliness of dose delivery																5	5%	7%
No need for shake test													6	9%	3%			
Helps since there is no vaccine vial monitor for freezing													2	0%	7%			
Reduces worry or stress for health workers													12	18%	7%			

Abbreviations: IS, immunization staff; DM, decision-makers.

The numbers in the table are the number of respondents mentioning each perceived benefit of the innovation. Respondents did not receive any pre-populated lists and so provided these benefits based on the information shared about each innovation. Respondents could provide as many benefits as they desired. The total number shows the total number of respondents mentioning each perceived benefit. The percentages show the proportion of all respondents in that group (n = 55 IS or n = 29 DM) mentioning each perceived benefit. Blank cells show the benefit was not mentioned by any respondent.

We indicate the perceived benefits of each innovation that were mentioned by most respondents using this key:

a Perceived benefit of the innovation selected by most respondents.

b Perceived benefit of the innovation selected by second-most respondents.

c Perceived benefit of the innovation selected by third-most respondents.

**Table 5:** Perceived benefits identified for the vaccine-agnostic innovations and number (%) of respondents mentioning the benefits—in-depth interviews of 84 total respondents composed of 55 immunization staff (IS) and 29 decision-makers (DM).

Potential benefit	Sharps injury protection syringes			Vaccine vial monitors with threshold indicators			Barcodes		
	n = 84	% of IS	% of DM	n = 84	% of IS	% of DM	n = 84	% of IS	% of DM
Easier to prepare and or use/eases logistics	31^b^	42%	28%	20	22%	28%			
Saves health care workers time	25	31%	28%	26^c^	33%	28%	30^b^	38%	31%
Improves vaccine coverage or vaccine reach							4	5%	3%
Enables delivery outside of a health facility/by less skilled personnel				24	29%	28%			
Reduces needle-stick injuries	75^a^	96%	76%						
Prevents vaccine damage/vaccine wastage due to suspected heat exposure				41^a^	55%	38%			
Allows vaccine to be kept out of the cold chain/reduces cold chain logistics				27^b^	35%	28%			
Improves potency/quality				12	11%	21%			
Improves waste disposal/reduce healthcare waste	29^c^	38%	28%						
Prevents reuse of syringes	3	4%	3%						
Prevents need to recap syringes	2	2%	3%						
Reduces worry or stress for health workers				4	7%	0%			
Improves ability to track information or have information about vaccines							48^a^	60%	52%
Aids tracking of adverse events following immunization or recalls							26^c^	40%	14%
Improves record keeping/monitoring of vaccines							22	0%	76%
Helps with legibility of label							8	9%	10%
Eases transferring information to patient files							4	7%	0%
Improves timeliness of dose delivery							3	5%	0%
Improves monitoring of vaccines for heat exposure				10	10	0			

Abbreviations: IS, immunization staff; DM, decision-makers.

The numbers in the table are the number of respondents mentioning each perceived benefit of the innovation. Respondents did not receive any pre-populated lists and so had to provide these benefits based on the information shared about each innovation. Respondents could provide as many benefits as they desired. The total number shows the total number of respondents mentioning each perceived benefit. The percentages show the proportion of all respondents in that group (n = 55 IS or n = 29 DM) mentioning each perceived benefit. Blank cells show the benefit was not mentioned by any respondent.

We indicate the perceived benefits of each innovation that were mentioned by most respondents using this key:

a Perceived benefit of the innovation selected by most respondents.

b Perceived benefit of the innovation selected by second-most respondents.

c Perceived benefit of the innovation selected by third-most respondents.

The perceived benefits identified for the vaccine-agnostic innovations were aligned with the main purpose or feature of the innovation (Table 5). For VVM-TIs, the benefit mentioned by the most respondents (41/84) was preventing vaccine damage/wastage of vaccines. For SIP syringes, the benefit mentioned by the most respondents (75/84) was reducing needle-stick injuries while for barcodes, the benefit mentioned by the most respondents (48/84) was improving the ability to track information or have information about vaccines.

#### 3.3.2 Perceived challenges of the innovations

For the vaccine-specific innovations, cost implications including overall costs or price per dose were most frequently mentioned by respondents as a perceived challenge associated with adoption of these innovations (Table 6). Cold chain volume implications and complexity of using each of the innovations were mentioned as perceived challenges associated with CPADs and dual-chamber delivery devices. The need for community sensitization was mentioned by many respondents as a perceived challenge of MAPs as the innovation may be less acceptable to vaccine recipients or caregivers given the novel vaccination technique. This challenge was also mentioned second-most frequently by respondents for SDIs, another innovation resulting in a novel vaccination technique. Complexity of using the delivery device innovations was also a challenge that tended to be mentioned by immunization staff across these innovations and training needs were mentioned as a challenge across most of these innovations by decision-makers.

**Table 6:** Perceived challenges facing the implementation of the vaccine-specific innovations and number (%) of respondents mentioning the challenges—in-depth interviews of 84 total respondents composed of 55 immunization staff (IS) and 29 decision-makers (DM).

Potential challenges	Compact, prefilled, autodisable devices			Dual-chamber delivery devices			Microarray patches			Solid dose implants			Freeze damage resistant liquid vaccines			Heat-stable/CTC qualified liquid vaccines		
	n = 84	% of IS	% of DM	n = 84	% of IS	% of DM	n = 84	% of IS	% of DM	n = 84	% of IS	% of DM	n = 84	% of IS	% of DM	n = 84	% of IS	% of DM
Needs community sensitization/less acceptable to parents/caregivers	10	18%	0%	8	13%	3%	22^a^	36%	7%	27^b^	45%	7%	4	5%	3%	6	11%	0%
Overall cost	20^c^	7%	55%	23^c^	13%	55%	19^b^	5%	55%	16	9%	38%	19^a^	13%	41%	19^a^	7%	52%
Cold chain volume	25^a^	24%	41%	25^b^	24%	41%	17^c^	20%	21%	17^c^	16%	28%	2	0%	7%			
Time required/complexity of using the technology	24^b^	31%	24%	27^a^	49%	0%	16	29%	0%	34^a^	51%	21%						
Training needs and or health care worker sensitization				9	0%	31%	11	0%	38%	9	0%	31%	5	0%	17%	7	0%	24%
Price per dose	9	0%	31%	7	13%	0%	8	0%	28%	8	0%	28%	8^b^	0%	28%	7	0%	24%
Concern about skin reactions or different absorption by skin type							6	9%	3%									
No indication that vaccine has been delivered							5	9%	0%									
Self-administration may be a challenge							5	9%	0%									
Safety concerns				4	7%	0%				11	20%	0%	4	5%	3%			
Concerns about potency													2	0%	7%			
Storage or logistics concerns										9	5%	21%						
May result in carelessness or confusion in vaccine management													6^c^	9%	3%	8^c^	9%	10%
Equipment needs										5	0%	17%						
Waste disposal	7	9%	7%							4	4%	7%						
Risk of vaccine wastage																8^c^	11%	7%
Availability or sustainability													2	4%	0%			
Concern about packaging and integrity of seals	7	9%	7%	5	9%	0%												
Concern that some of the dose may not be delivered	7	13%	0%	0														
Concerns about needle size				4	4%	7%												
Complexity of CTC protocol																9^b^	13%	7%
Not enough CTC qualified vaccine																5	9%	0%
Requirement of additional logistics																3	0%	10%
Number of days out of cold chain needs to be higher																3	0%	10%

Abbreviations: IS, immunization staff; DM, decision-makers.

The numbers in the table are the number of respondents mentioning each perceived challenge of the innovation. Respondents did not receive any pre-populated lists and so had to provide these challenges based on the information shared about each innovation. Respondents could provide as many challenges as they desired. The total number shows the total number of respondents mentioning each perceived challenge. The percentages show the proportion of all respondents in that group (n = 55 IS or n = 29 DM) mentioning each perceived challenge. Blank cells show the challenge was not mentioned by any respondent.

We indicate the perceived challenges of each innovation that were mentioned by most respondents using this key:

a Perceived challenge of the innovation selected by most respondents.

b Perceived challenge of the innovation selected by second-most respondents.

c Perceived challenge of the innovation selected by third-most respondents.

As shown in Table 7 focusing on challenges for the vaccine-agnostic innovations, the need to procure appropriate equipment was the most mentioned challenge for barcode adoption followed by the complexity of using the innovation. Cost implications were most frequently mentioned as a challenge for SIP syringes and VVM-TIs. The second-most mentioned challenges were time required/complexity of use for SIP syringes and training requirements for VVM-TIs.

**Table 7:** Perceived challenges identified for the vaccine-agnostic innovations and number (%) of respondents mentioning the challenges—in-depth interviews of 84 total respondents composed of 55 immunization staff (IS) and 29 decision-makers (DM).

Potential challenges	Sharps injury protection syringes			Vaccine vial monitors with threshold indicators			Barcodes		
	n = 84	% of IS	% of DM	n = 84	% of IS	% of DM	n = 84	% of IS	% of DM
Overall cost	12^a^	7%	28%	11^a^	5%	28%	12^c^	11%	21%
Time required/complexity of using the technology	9^b^	16%	0%				21^b^	38%	0%
Training needs and/or health care worker sensitization	7	0%	24%	9^b^	0%	31%	11	4%	31%
Price per dose	8^c^	0%	28%	6^c^	0%	21%	2	0%	7%
Equipment needs							53^a^	71%	48%
Risk of vaccine wastage				5	9%	0%			
Provides no indication of freezing				1	2%	0%			
Only useful when using CTC strategy				1	0%	3%			
Internet connectivity or power supply issues							10	15%	7%
Feasibility at service delivery level							5	0%	17%
Data security concerns							1	2%	0%
Availability or sustainability	2	4%	0%						

Abbreviations: IS, immunization staff; DM, decision-makers.

The numbers in the table are the number of respondents mentioning each perceived challenge of the innovation. Respondents did not receive any pre-populated lists and so had to provide these challenges based on the information shared about each innovation. Respondents could provide as many challenges as they desired. The total number shows the total number of respondents mentioning each perceived challenge. The percentages show the proportion of all respondents in that group (n = 55 IS or n = 29 DM) mentioning each perceived challenge. Blank cells show the challenge was not mentioned by any respondent.

We indicate the perceived challenges of each innovation that were mentioned by most respondents using this key:

a Perceived challenge of the innovation selected by most respondents.

b Perceived challenge of the innovation selected by second-most respondents.

c Perceived challenge of the innovation selected by third-most respondents.

#### 3.3.3 Vaccines for which the innovations could be most useful

Table 8 shows the total number of respondents mentioning the vaccines or class of vaccines for which they believe each innovation would be most useful. Dual-chamber delivery devices, MAPs, and SDIs were identified as innovations that would be most useful for measles-containing and Bacillus Calmette-Guérin vaccines. This is consistent with the observation that they would remove the need for reconstitution of lyophilized vaccines and, due to their single-dose format, avoid missed opportunities due to reluctance to open a multidose vial and prevent vaccine wastage of unused reconstituted, preservative-free vaccine. MAPs and SDIs were also mentioned as most useful for inactivated poliovirus vaccine (IPV), pentavalent, and HPV vaccines. CPADs were perceived as being most useful for IPV, pentavalent, and pneumococcal conjugate vaccine even though they would not address the highest ranked challenges of vaccine ineffectiveness or wastage due to heat or freeze exposure identified for IPV and pentavalent vaccine in the second online survey (Table 3). Freeze damage resistant liquid formulations were identified as being most useful for pentavalent vaccine, IPV, and tetanus toxoid-containing vaccine. Heat-stable/CTC qualified liquid vaccines were perceived as being most useful for HPV, pentavalent, and IPV.

**Table 8:** Number of respondents mentioning the vaccines or class of vaccines for which the innovations would be most useful—in-depth interviews of 84 total respondents.

Vaccine name or description	Compact, prefilled, autodisable devices	Dual-chamber delivery devices	Microarray patches	Solid dose implants	Freeze damage resistant liquid vaccines	Heat-stable/controlled temperature chain qualified liquid vaccines
Measles-containing vaccine	11	55 a	34 a	33^a^		13
Bacille Calmette-Guérin (BCG)^1^	16	54^b^	21^b^	17^b^		8
Inactivated poliovirus vaccine^2^	37^a^	1	18^c^	11	38^b^	22^c^
Pentavalent (DTP-HepB-Hib) vaccine^3^	32^b^		17	17^b^	50 a	23^b^
Human papillomavirus vaccine	13		17	10	21	27 a
Hepatitis B birth dose vaccine	18		15	8	27	7
Tetanus toxoid-containing vaccine (other than pentavalent)	14	3	10	7	33^c^	13
Pneumococcal conjugate vaccine	19^c^		7	6	26	11
Japanese encephalitis vaccine	3	8	2	5		
Rabies (lyophilized) vaccine, post exposure		1	3			
Yellow fever vaccine^4^	1	16^c^	6	3		3
Meningococcal conjugate vaccine	9	10	6	2		15
Influenza vaccine			1			1
Typhoid conjugate vaccine			1			4
Oral rotavirus vaccine, liquid products only^5^	2	2	1		6	5
Oral poliovirus vaccine (or non-specified polio vaccine)	1			2		19
Oral cholera vaccine						5
Malaria vaccine^6^						1
All EPI vaccines	5	1	6	3		6
Vaccines given to older children (or booster doses)			1			
Vaccines given to adults				1		
Parenteral vaccines	1		10	8		
Subcutaneous vaccines			1	2		
Oral vaccines				1		
Liquid vaccines	8	1	1		8	3
Vaccines that need to be reconstituted	1	11	2	4	1	
Multidose presentations	1	1	2	1	2	
Single-dose presentations		1				1
Freeze-sensitive vaccines		1			16	
Diluents that are freeze sensitive					1	
Heat-sensitive vaccines						10
Heat-stable vaccines						1
Vaccines used in campaigns				1		3
Vaccines administered by lay health workers	1	1				
No specific vaccine	1		2	1		1
Not recommended for current vaccines	1			3		1
Medications (rather than vaccines)			1	1		

Abbreviations: IS, immunization staff; DM, decision-makers.

The numbers in the table are the number of respondents mentioning the vaccines or class of vaccines for which each of the vaccine-specific innovations would be most useful. Respondents did not receive any pre-populated lists and so had to provide these vaccines based on the information shared about each innovation. Respondents could provide as many vaccines as they desired. The total number shows the total number of respondents mentioning each vaccine or class of vaccines for which each innovation could be most useful. Blank cells show the challenge was not mentioned by any respondent.

We indicate the vaccines or class of vaccines for each innovation that were mentioned by most respondents using this key:

a Vaccines or class of vaccines mentioned for the innovation by most respondents.

b Vaccines or class of vaccines mentioned for the innovation by second-most respondents.

c Vaccines or class of vaccines mentioned for the innovation by third-most respondents.

1 Mentioned by respondents but was not assessed for use with heat-stable liquid/CTC qualified vaccines and SDIs by VIPS due to concerns about technical feasibility.

2 Mentioned by respondents but was not assessed for use with dual-chamber delivery devices by VIPS due to concerns about technical feasibility.

3 Mentioned by respondents but was not assessed for use with MAPs by VIPS due to concerns about technical feasibility.

4 Mentioned by respondents but was not assessed for use with heat-stable liquid/CTC qualified vaccines by VIPS due to concerns about technical feasibility.

5 Mentioned by respondents but was not assessed for use with dual-chamber delivery devices by VIPS due to concerns about technical feasibility.

6 Mentioned by respondents but was not assessed for use with heat-stable liquid/CTC qualified vaccines by VIPS due to concerns about technical feasibility.

The respondents were also asked about the immunization delivery setting and the target population for which the innovations would be most useful for. However, most respondents said the innovations were useful for all settings and all eligible vaccine target populations and did not generally prioritize one setting or population over another. These results are not reported in the tables.

#### 3.3.4 Additional information gathered about the innovations

While answering the open-ended questions, respondents provided feedback about some of the innovations beyond their benefits and challenges. For MAPs, immunization staff mentioned their preference for smaller MAPs without applicators. Similarly, for SDIs, respondents reported that they preferred the version with a disposable applicator instead of the one with a reusable applicator. Respondents also stated that they desired innovations that could combine multiple vaccines to reduce the number of vaccinations. For heat-stable/CTC qualified liquid vaccines, decision-makers provided general feedback that the number of minimum days in CTC use needed to be longer than the current three days and should be at least seven days. For SIP syringes, decision-makers preferred the version with a retractable needle over the one with a needle shield, due to safety concerns given that the version with the needle shield requires manual manipulation too close to the needle; the shield getting in the way during injections was also a concern. They also commented that if SIP syringes were procured, they should be available to all health programs to avoid syringe diversion to health programs other than immunization. Respondents also suggested combining innovations such as heat-stable/CTC qualified liquid vaccines with VVM-TIs, CPADs with heat-stable vaccines, and CPADs with SIP features.

#### 3.3.5 Ranking of the innovations

**Fig. 1:**
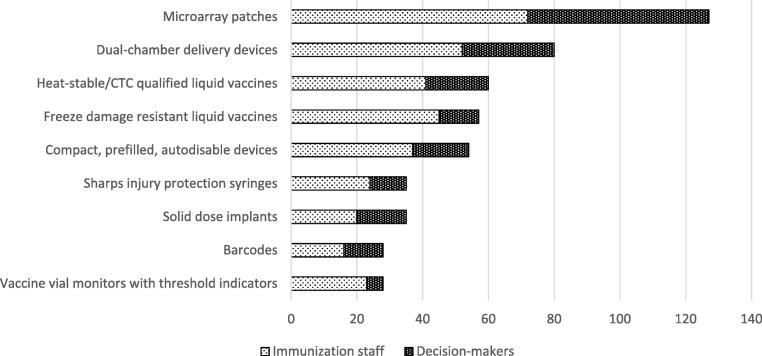
Weighted ranking of the innovation—results from in-depth interviews of 84 total respondents composed of 55 immunization staff (IS) and 29 decision-makers (DM).

As shown in Fig. 1 displaying the weighted ranking of the innovations, respondents suggested that MAPs, dual-chamber delivery devices, and heat-stable/CTC qualified liquid vaccines would have the greatest impact in helping address their immunization program’s current challenges. The ranking of innovations was broadly consistent between decision-makers and immunization staff. These results also align with the results of the first online survey. For instance, both MAPs and dual-chamber delivery devices can prevent missed opportunities as single-dose presentations, which was selected as the most desirable vaccine attribute for routine facility-based immunization. The ability to withstand heat exposure, which can be achieved through heat-stable/CTC qualified liquid vaccines, was the most desired vaccine attribute for outreach and campaign settings.

#### 3.3.6 Summary of the in-depth interviews to evaluate VIPS short-listed innovations

The learnings from the in-depth interviews provided critical perspectives from country stakeholders on the possible benefits and challenges associated with each innovation, as well as where the greatest potential and interest lies. Detailed information obtained on the perceived benefits and challenges and the most useful vaccine-innovation pairings will also inform follow-on activities for the prioritized innovations, and could inform continued development of all assessed innovations, given that critical feedback was obtained on issues, such as product profile considerations, costs considerations, and training requirements for product introduction.

### 3.4 Limitations of the surveys

A key limitation of the two online surveys was the low participation from respondents in non-Gavi-supported, middle-income countries and the Americas, Eastern Europe and the Eastern Mediterranean, despite targeted efforts to elicit responses from these regions. A few countries also had proportionately higher response rates to the online surveys than others. Due to the online format, the reach of the survey was also limited by access to suitable devices and a stable internet connection. The second online survey’s design was also limited by the vaccine-specific challenges not being evaluated for different settings or delivery strategies (i.e., routine facility-based vs. outreach vs. campaigns) in the interest of keeping the survey length manageable and maximizing the completion rate. This prevented a more comprehensive comparison to results from the first survey. A limitation of the in-depth interviews was that the interviews were only conducted in six Gavi countries (five from Africa and one from South East Asia) due to limited resources and time for partners to conduct or EPI programs to participate in the in-person, in-depth interviews. While the three consultations included responses from many countries, we do not report results disaggregated by country or region as these consultations were designed as global surveys and not powered for country- or regional-level sub-analyses.

## 4 Conclusions

Understanding countries’ immunization challenges that could be addressed through vaccine product innovations was a foundation of the VIPS process, and the insights generated through the three consultations with varied country stakeholders informed the VIPS prioritization process. The first phase of VIPS utilized an analytical framework with specific indicators to assess an initial list of 24 innovation types that were short-listed to 9 innovations based on their breadth of potential public health benefits or unique benefits and applicability to several vaccines. The results from the first survey on general immunization barriers were used to provide a qualitative weighting to the indicators that addressed the most important barriers identified by countries. In the second VIPS phase, the nine short-listed innovations were further assessed with representative vaccines, based on a more complete analytical evaluation framework. Innovations were prioritized based on indicators addressing the most important challenges identified by countries for a majority of vaccines (from the second survey) and based on the level of interest (innovation’s ranking) from country stakeholders on these innovations (from the in-depth interviews).

The results of these three country consultations strongly influenced the final VIPS prioritization of MAPs, heat-stable/CTC qualified vaccines, and barcodes on vaccine primary packaging as they were an important component of a broader evaluation of each innovation’s potential impact. Factors considered in the broader evaluation of the innovations included potential public health benefit and impact on coverage and equity, safety, total costs, and the environment, as well as each innovation’s technology readiness and commercial feasibility. The VIPS Alliance also desired to prioritize a balanced portfolio of different innovation profiles, (e.g., in terms of risk to success based on the stage in the product development pathway and required resources to bring the innovation to market or scale) [14].

Two of the three prioritized innovations at the end of the VIPS process, MAPs and heat-stable/CTC qualified vaccines, were identified in the in-depth interviews as two of the top three innovations that could have the greatest impact in helping address current immunization program challenges. Prioritization of these innovations aligns with the outcomes of the two online surveys since they both address the most challenging immunization barriers by virtue of their valuable vaccine product attributes. Some of these attributes for MAPs include being a single-dose presentation, which would reduce missed opportunities for vaccination and vaccine wastage, potential for enhanced thermostability thereby facilitating outreach, and not needing reconstitution hence avoiding reconstitution-related safety issues. For heat-stable/CTC qualified vaccines, these attributes include ability to withstand heat exposure, and minimize cold chain requirements during outreach.

Regarding the prioritization of barcodes, while the in-depth interview participants did not rate them highly against the other delivery and formulation innovations, the second online survey respondents expressed strong interest in transitioning from paper-based to electronic systems for patient vaccination records and vaccine inventories in which barcodes can play a facilitative role. Their prioritization also is intended to support the ongoing efforts of UNICEF, Gavi, and other stakeholders to improve traceability of vaccine products, including COVID-19 vaccines, for LMICs.

Although dual-chamber delivery devices were ranked second (after MAPs) in the in-depth interviews, they were not prioritized by VIPS in order to achieve a diversified portfolio. Both dual-chamber delivery devices and MAPs offer similar benefits (e.g., reducing missed opportunities and avoiding reconstitution errors), and both also face significant technical and manufacturing challenges and are in early stages of development. However, there is more catalytic work, including investments, underway for MAPs that can be harnessed to move this innovation forward.

The final VIPS prioritization is an important first step towards driving product innovation to better meet LMICs’ needs, but significant work is still needed to achieve uptake of any innovation as stated country preferences do not imply country adoption when the innovation becomes available for use and there are numerous other barriers preventing adoption and scale up. To better understand these barriers and identify factors impacting country adoption of innovations, the VIPS Alliance analyzed four commercially available vaccine-product-innovations and augmented the evaluation with interviews with 17 experts. The findings are summarized in a VIPS accompanying article titled *Strategies for vaccine-product innovation: creating an enabling environment for product development- to-uptake in low- and middle-income countries* [15]. The article also highlights actions that should be undertaken in parallel to product development to incentivize sustainable investment and prepare the pathway for uptake and impact.

Recognizing the substantial work that lies ahead, the VIPS Alliance is now developing and implementing end-to-end strategies for each of the three prioritised innovations, including 5-year action plans to accelerate their development and uptake. Activities in the action plans [8] include prioritizing vaccine applications for development, assessing the innovations’ full economic value and health impact, and understanding willingness-to-pay, clarifying potential demand, identifying and addressing research gaps and needs for implementation research, defining investment cases and the need for new procurement/financing mechanisms, as well as understanding the need for additional push funding. As one of the key components of these 5-year action plans, the VIPS Alliance will ensure sustained engagement with country- and regional-level stakeholders, which will be essential to clarify and confirm key assumptions in terms of use case scenarios, product preferences, potential demand, and willingness to pay for these innovations, and ensure that country priorities and preferences are central to design and investment in these innovations and ensure successful programmatic impact.

## Declaration of Competing Interest

The authors declare the following financial interests/personal relationships which may be considered as potential competing interests: The authors confirm that the work submitted for consideration has not been published previously and is not under consideration for publication elsewhere. The submission has been approved by all authors. If the manuscript is accepted by this journal, it will not be published elsewhere in the same form.

## Footnotes

Appendix A Supplementary data to this article can be found online at https://doi.org/10.1016/j.vaccine.2021.08.024.
