# Supplementary figures and images for: Vaccine innovation prioritisation strategy: Findings from three country-stakeholder consultations on vaccine product innovations
Source: Vaccine. 2021 Dec 3;39(49):7195–207. doi: 10.1016/j.vaccine.2021.08.024 (PMC8657797; doi:10.1016/j.vaccine.2021.08.024)

**Supplementary Figure 1.** Completion by organization for the first online survey (n=442).


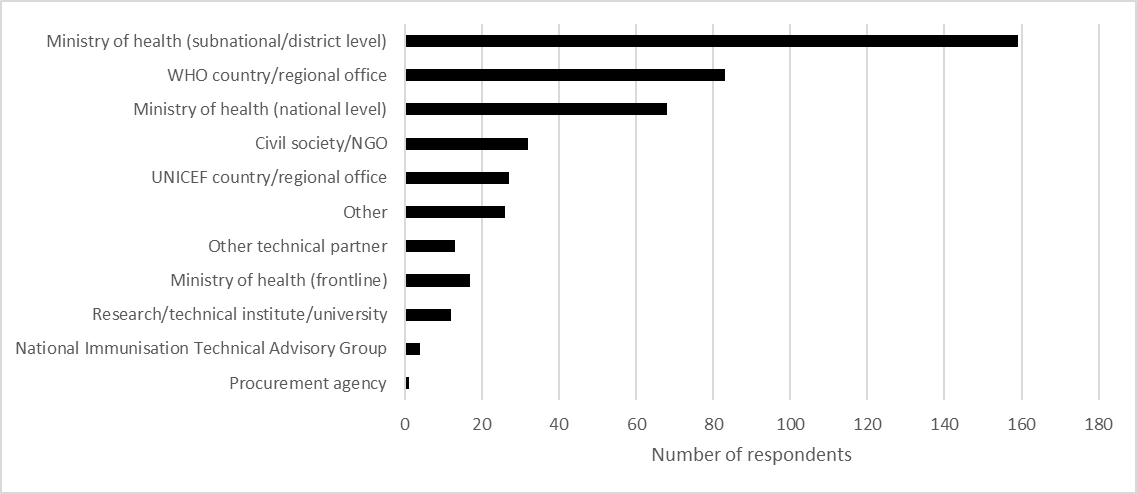

Supplement: Supplementary data 1 [file mmc1.docx]

**Supplementary Figure 2.** Completion by organization for the second online survey (n=220).

**
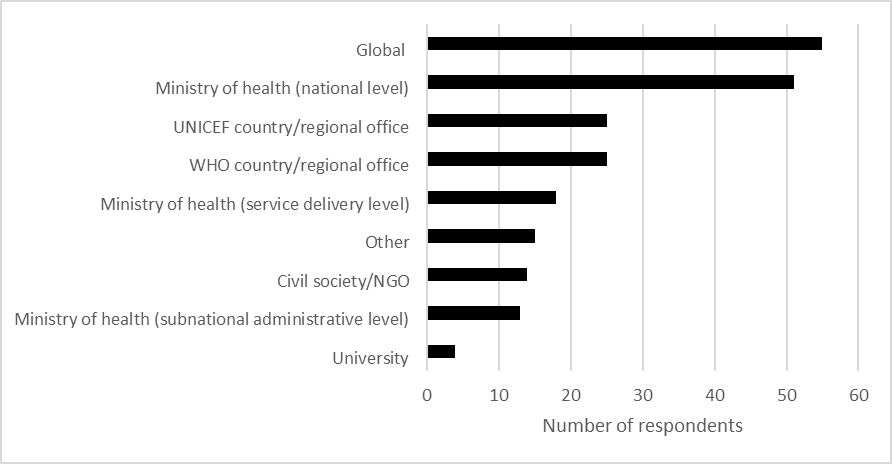
**

Supplement: Supplementary data 2 [file mmc2.docx]
