# Supplementary material for: Vaccine innovation prioritisation strategy: Findings from three country-stakeholder consultations on vaccine product innovations
Source: Vaccine. 2021 Dec 3;39(49):7195–207. doi: 10.1016/j.vaccine.2021.08.024 (PMC8657797; doi:10.1016/j.vaccine.2021.08.024)
Supplement: Supplementary data 3 [file mmc3.docx]

**Supplementary Table 1.** Online survey on general immunization barriers survey questions.

| **Out of 18 implementation barriers, please select the top five barriers that prevent improvements in coverage and equity in each use setting (routine facility-based, outreach, campaigns).**   - Wastage due to heat exposure - Vaccine transport damage - Mistrust in health care worker - Wastage due to freeze exposure - Missed opportunities due to complexity of vaccine preparation/administration - Damage due to inappropriate storage - Shortages of commodities required for administration - Fear of injections and needles - Social barriers - Financial barriers - Insufficient cold chain capacity - Discomfort after vaccination - Lack of training and skills - Geographic barriers - Shortages due to poor tracking - Lack of available health care workers - Inadequate infrastructure for storage (excluding cold chain capacity) - Missed opportunities due to reluctance to open multidose vials |
| --- |
| **Out of 15 vaccine product attributes, please select the top five most valuable attributes that could help address the implementation barriers that you outlined earlier in the survey for each use setting (routine facility-based, outreach, campaigns).**   - Ability to withstand freeze exposure - Suitable for self-administration - Reduced risk of needle-stick injury - Reduced risk of incorrect delivery - Reduced risk of incorrect preparation - Reduced risk of vaccine contamination - Suitable for controlled temperature chain use - Minimal number of separate components necessary for administration - Reduced space required for storage and transport - Delivery aligned with existing schedules - Suitable for administration by less trained personnel - Easier to use reducing time for preparation and administration - Acceptable to patients/caregivers - Ability to withstand heat exposure - Presentation reducing missed opportunities |
| ***What other valuable vaccine product attribute would you like to see and for which setting would it apply?*** |
