# Supplementary material for: Vaccine innovation prioritisation strategy: Findings from three country-stakeholder consultations on vaccine product innovations
Source: Vaccine. 2021 Dec 3;39(49):7195–207. doi: 10.1016/j.vaccine.2021.08.024 (PMC8657797; doi:10.1016/j.vaccine.2021.08.024)
Supplement: Supplementary data 4 [file mmc4.docx]

Supplementary Table 2. Online survey on vaccine-specific immunization challenges survey questions.

| **Vaccine expertise** |
| --- |
| **Please select each vaccine below for which you have experience and an in-depth understanding of its vaccination strategy and use (for example, you understand the issues related to storage, transport, preparation, and administration of this vaccine type).**   - Pentavalent (DTP-HepB-Hib) vaccine (Penta) - Measles-containing vaccine (MCV) - Meningitis A vaccine (MenA) - Hepatitis B birth dose vaccine (HepB BD) - Human papillomavirus (HPV) vaccine - Inactivated poliovirus vaccine (IPV) - Lyophilized rabies vaccine (post-exposure indication) - Liquid oral rotavirus vaccine (rota) - Typhoid conjugate vaccine (TCV) - Yellow fever (YF) vaccine - None of the above   [THE “VACCINE EXPERTISE” QUESTION WILL IDENTIFY WHICH VACCINES THE RESPONDENT HAS EXPERIENCE WITH AND THEN THEY WILL ONLY BE ASKED QUESTIONS ABOUT THOSE VACCINES. IF “NONE OF THE ABOVE” IS SELECTED, THEN THE RESPONDENT WILL SKIP TO THE “ELECTRONIC INVENTORY OF VACCINES” SECTION.] |
| **Vaccine-specific questions** |
| **Based on your expertise, please review the following statements describing immunization challenges that could be solved by innovations and select the challenges that apply to [vaccine name]. Please check all that apply.**   - Administration of the vaccine is painful for vaccine recipients, which reduces acceptability. - The vaccine is difficult to prepare, which takes time during the immunization session and requires an appropriate level of skill. - Exposure of the vaccine to freezing temperatures can damage the vaccine resulting in delivery of ineffective vaccine or wastage. - Exposure of the vaccine to heat can damage the vaccine resulting in delivery of ineffective vaccine or wastage. - The need to keep the vaccine in the cold chain during outreach is a challenge. - Vaccine wastage or missed opportunities can occur because the vaccine is in a multidose vial without preservative. - Because the vaccine is in a multidose vial, contamination issues can occur. - The need to reconstitute the vaccine can result in safety issues due to vaccine contamination or use of wrong diluent. - Needle-stick injuries can occur during preparation, delivery, and/or disposal of sharps used for reconstitution or injection. - It is difficult to deliver the vaccine to the correct injection depth. - The waste disposal practices for the vaccine and/or accompanying delivery device have a particularly negative impact on the environment. - None of the above. - **Other** (please specify). |
| **Please select the three most important challenges out of those you have identified for [vaccine name]. if you selected fewer than three challenges, please select all the challenges you identified.**  [ONLY THE CHALLENGES SELECTED IN THE PREVIOUS QUESTION APPEAR IN THIS QUESTION AS THE ANSWER OPTIONS USING ANSWER PIPING IN SURVEYMONKEY.]   - ↪ *Administration of the vaccine is painful for vaccine recipients, which reduces acceptability.* - ↪ *The vaccine is difficult to prepare, which takes time during the immunization session and requires an appropriate level of skill.* - ↪ *Exposure of the vaccine to freezing temperatures can damage the vaccine resulting in delivery of ineffective vaccine or wastage. [Question is asked for freeze-sensitive vaccines only.]* - ↪ *Exposure of the vaccine to heat can damage the vaccine resulting in delivery of ineffective vaccine or wastage. [Question is asked for heat-sensitive vaccines only.]* - ↪ *The need to keep the vaccine in the cold chain during outreach is a challenge.* - ↪ *Vaccine wastage or missed opportunities can occur because the vaccine is in a multidose vial without preservative. [Question is asked for vaccines in multidose vial without preservative only.]* - ↪ *Because the vaccine is in a multidose vial, contamination issues can occur. [Question is asked for liquid vaccines only.]* - ↪ *The need to reconstitute the vaccine can result in safety issues due to vaccine contamination or use of wrong diluent. [Question is asked for lyophilized vaccines only.]* - ↪ *Needle-stick injuries can occur during preparation, delivery, and/or disposal of sharps used for reconstitution or injection. [Question is asked for injectable vaccines only.]* - ↪ *It is difficult to deliver the vaccine to the correct injection depth. [Question is asked for injectable vaccines only.]* - ↪ *The waste disposal practices for the vaccine and/or accompanying delivery device have a particularly negative impact on the environment.* - ↪ *None of the above.* - ↪ *[Insert text from other].* |
| **Please rank the challenges you have identified for [vaccine name] in order of importance—1 being the most important challenge and 3 being the least important challenge. Simply move the rows to place (rank) them in order of importance.**  [ONLY THE MOST IMPORTANT CHALLENGES SELECTED IN THE PREVIOUS QUESTION APPEAR IN THIS QUESTION AS THE ANSWER OPTIONS FOR RANKING IN THIS QUESTION USING ANSWER PIPING IN SURVEYMONKEY. THE RESPONDENT WILL BE ABLE TO MOVE THE ITEMS IN THE LIST OF CHALLENGES TO QUICKLY RANK THEM FROM MOST TO LEAST IMPORTANT. INSTRUCTION WILL BE GIVEN ON HOW TO DO THIS.]   \| 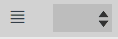*[text from challenge 1]* \| \| --- \| \| *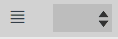[text from challenge 2]* \| \| *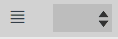[text from challenge 3]* \| |
| **Please explain your selection of the most important challenge (ranked as 1) to achieving your immunization program’s goals for [vaccine name].** |
| **[Optional Question] Please let us know if you have suggestions to improve the current [vaccine name] product to address the challenge you identified as most important.** |
| **Electronic inventory of vaccines** |
| **Does the public immunization program in the country where you primarily work use an electronic system (for example, computer, tablet, smartphone) to inventory vaccines?**   - Yes - No - Do not know |
| **Would your public immunization program benefit by transitioning from a paper-based system to an electronic system (for example, using computers, tablets, or smartphones) for the inventory of vaccines?**   - Yes - No |
| **Electronic patient vaccination records** |
| **Does the public immunization program in the country where you primarily work use electronic patient vaccination records (for example, is it using computers, tablets, or smartphones)?**   - Yes - No - Do not know |
| **Would your public immunization program benefit by transitioning from a paper-based system to an electronic system for patient vaccination records?**   - Yes - No |
