# Supplementary material for: Vaccine innovation prioritisation strategy: Findings from three country-stakeholder consultations on vaccine product innovations
Source: Vaccine. 2021 Dec 3;39(49):7195–207. doi: 10.1016/j.vaccine.2021.08.024 (PMC8657797; doi:10.1016/j.vaccine.2021.08.024)
Supplement: Supplementary data 5 [file mmc5.docx]

**Supplementary Table 3.** Questions included in the phase 2 in-depth interviews.

| **Question** | **Question targeted to:** | **Innovations included** |
| --- | --- | --- |
| In your opinion, how could *[innovation name]* help your immunization program meet its goals? | All respondents: Decision-makers and immunization staff | All nine innovations:   - Compact prefilled autodisable devices - Dual-chamber delivery devices - Microarray patches - Solid dose implants - Sharps injury protection syringes - Freeze damage resistant liquid vaccines - Heat-stable liquid/controlled temperature chain qualified vaccines - Vaccine vial monitors with threshold indicators - Barcodes on vaccine primary containers |
| Would you consider recommending purchase of *a vaccine in a (or with a) [innovation name]* if it were available? (Yes or No). | Decision-makers | All nine innovations |
| Besides the need for training, can you foresee any new challenges that *[innovation name]* would introduce if adopted? | Immunization staff | All nine innovations |
| Are there any specific vaccines for which you think *[innovation name]* would be particularly useful, and why? For what immunization strategy would this apply and for what target population? | All respondents: Decision-makers and immunization staff | - Compact prefilled autodisable devices - Dual-chamber delivery devices - Microarray patches - Solid dose implants - Freeze damage resistant liquid vaccines - Heat-stable liquid/controlled temperature chain qualified vaccines |
| Select the top three innovations that you believe would have the greatest impact in helping address your immunization program’s current challenges. | All respondents: Decision-makers and immunization staff | All nine innovations |
